# Raised serum levels of IGFBP-1 and IGFBP-2 in idiopathic pulmonary fibrosis

**DOI:** 10.1186/s12890-016-0249-6

**Published:** 2016-05-23

**Authors:** J. Guiot, B. Bondue, M. Henket, J. L. Corhay, R. Louis

**Affiliations:** Pneumology Department, CHU Liège, Domaine universitaire du Sart-Tilman, B35, B4000 Liège, Belgium; Pneumology Department, Erasme University Hospital, Université Libre de Bruxelles, Route de Lennik, 808, B1070 Brussels, Belgium

**Keywords:** Idiopathic pulmonary fibrosis, Pulmonary fibrosis, Insuline-like growth factors, Insulin like growth factor binding proteins

## Abstract

**Background:**

Idiopathic pulmonary fibrosis (IPF) is a chronic lung disorder of unknown origin, which ultimately leads to death. Several growth factors such as IGFs (insulin-like-growth factor) and IGFBPs (insulin like growth factor binding proteins) seem to take part to the pathogenesis. We evaluated IGFs and IGFBPs in serum from patients with IPF and healthy subjects including 24 untreated IPF and 26 IPF receiving anti-fibrotic therapy and to compare them with healthy subjects.

**Methods:**

Serum of 50 idiopathic pulmonary fibrosis and 55 healthy subjects (HS) were analysed by ELISA for IGFs and IGFBPs, TGF-β and KL-6, the latter being tested as positive control in IPF.

**Results:**

Serum levels of IGFBP-1 and IGFBP-2 and KL-6 were significantly higher in the IPF group than in the healthy subjects (*p* < 0.05, *p* < 0.001 and *p* < 0.0001 respectively) while the picture was inversed regarding IGFs. By contrast there was no significant difference between the groups with respect to TGF-β. IGFBP-2 was significantly reduced in the patients with specific anti-fibrotic therapy pirfenidone and nintedanib compared to untreated patients (*p* < 0.05) but still significantly elevated in comparison to HS (*p* < 0.001).

**Conclusion:**

Serum IGFBP-1 and −2 are increased in idiopathic pulmonary fibrosis and IGFBP-2 may be reduced by anti-fibrosing therapy. IGFBPs may be promising biomarkers in IPF.

**Electronic supplementary material:**

The online version of this article (doi:10.1186/s12890-016-0249-6) contains supplementary material, which is available to authorized users.

## Background

Idiopathic pulmonary fibrosis (IPF) is a complex diagnosis and pathology requiring a multidisciplinary approach. This fibrotic disease has a poor prognosis and requires a specific and early appropriate therapy [1–3]. In this context several biomarkers (such as surfactant protein A or SP-A, the Krebs von den Lungen 6 or KL-6, Ig A and periostin [4–8] were studied without much success to be discriminant as early diagnostic biomarkers to identify IPF out of different interstitial lung diseases (ILDs). Nevertheless when taken together they provided convincing evidence that changes in blood proteins (KL-6, SP-A, MMP-7, CCL-18, among others) or cells (fibrocytes and T-cell subpopulations) are indicative in IPF and may somewhat predict outcome of the disease [9].

The transforming growth factor beta (TGF-β) has long been known to be involved in the pathophysiology of the IPF [1]. TGF-β is a stimulus for pulmonary fibrogenesis by its activity in the control of the remodelling of the extracellular matrix. TGF-β is also known to be involved in the fibroblast activity.

Besides TGF-β, there has been recently a growing interest for the axis IGFs/IGFBPs in fibrosing process. A recently published study has shown a link between the production of TGF-β and the production of insulin-like growth factor binding protein-2 (IGFBP-2) [10] known to be related to IPF [11] within myofibroblasts cells derived from the lung. Moreover, IGFBP-2 was found at significantly higher level in the bronchoalveolar lavage of children with interstitial lung disease [12] relative to healthy subjects.

IGFBP-2 is also overexpressed in response to a deterioration of the lung parenchyma [13–15]. This overexpression is mainly perinuclear and proves to be a potential factor of fibroblast proliferation [16–19]. The IGFBP-2 protein is part of a group (IGFBPs) known to be involved in the regulation of insulin-like growth factor (IGF). IGF-1 and IGF-2 play an important role in the growth, differentiation and cellular metabolism [20, 21]. Signalling pathway of IGF consisted of two isoforms of IGF (−1 and −2), two types of receptors, six binding proteins with high affinity for IGFs (IGFBPs) and four binding proteins with low affinity for IGFs (or related protein IGFBP: IGFBPrp) [11, 22, 23]. The IGFBPs bind IGFs and can increase their half-life, alter their function (in potentiating or inhibiting it), or facilitate their passage to the target tissues [20, 24]. Activity of IGFBP-2 is controlled on one hand by its secretion and on the other hand by its proteolysis. Proteolysis of IGFBP-2 is made by several groups of proteolytic enzymes such as pappalysines, kallikreins, metalloproteases or the plasmin [25].

There has been recent pharmacological progress in the treatment of IPF. Drugs such as pirfenidone and nintedanib have been shown to slow down the decline in lung volume that accompanies the lung fibrosing process [2].

We focused our study on the serum measurement of several growth factors including IGFs and IGFBPs in order to identify a potential new biomarker in IPF and sought to determine whether their levels might be influenced by recently developed anti-fibrotic therapy.

## Methods

### Subject characteristics

**Fig. 1:**
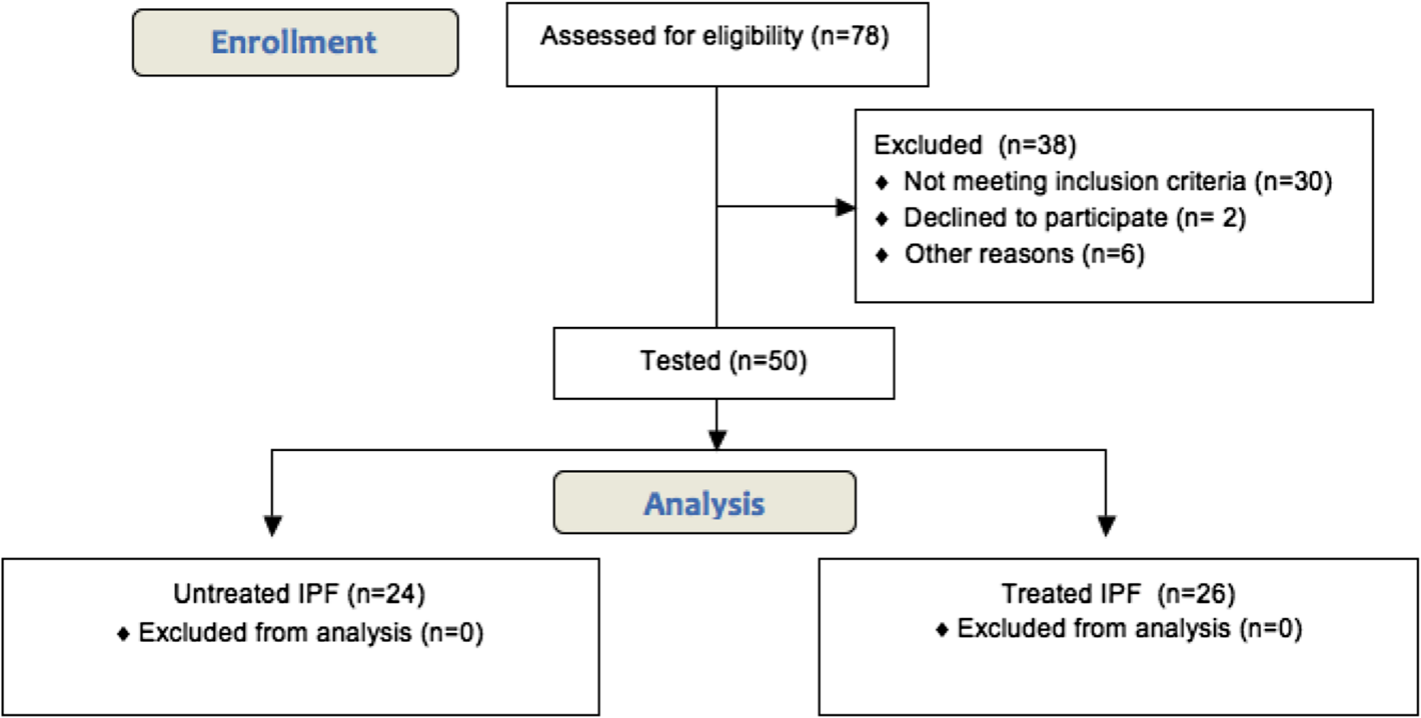
CONSORT flow chart

We prospectively recruited patients from our ambulatory care policlinic at CHU Liege and Erasme University Hospital of Brussels. The patients were divided into 2 groups. The first group was the group of patients with untreated IPF (*n* = 24). The second group is a group of treated IPF patients with a specific therapy (*n* = 26). The diagnosis of (definite) IPF was made according to the international recommendations of the ATS [1] using the respiratory function test, HRCT scan (probable UIP pattern), bronchoalveolar lavage (when available), as well as the clinical history of the patient. We excluded all other causes of interstitial lung disease (such as asbestosis, hypersensitivity pneumonitis, pneumonia associated with connective tissue disease or toxic pneumonitis). We combined the different results for the diagnosis. All cases were discussed in a multidisciplinary group about interstitial lung diseases composed of a pulmonologist, a specialist in pulmonary rehabilitation, a rheumatologist, a radiologist, a pathologist and a specialist in occupational medicine. Sixteen patients underwent a surgical biopsy and seven patients trans-bronchial cryobiopsies. Twenty-six patients benefit from a specific treatment of IPF (pirfenidone (*n* = 17) or nintedanib (*n* = 9)) Fig. 1. We also recruited healthy subjects by advertisement in our policlinic waiting room. They all denied any respiratory disease and had normal spirometric values with FEV1 > 80 % predicted and FEV1/FVC ratio > 70 %.

The protocol was approved by the ethics committee of CHU of Liège, and all subjects gave written consent before their enrollment (Belgian number : B707201422832 ; ref : 2014/302).

### Peripheral blood puncture

Venous blood was collected in Vacutainer tubes from an antecubital site immediately when controls and patients were included in the study. Blood cell values included white blood cell count, the differential leukocyte count, fibrinogen and C-reactive protein (CRP) levels were determined by the routine hospital laboratory.

### Biomarkers measurements in serum

We analysed several biomarkers assumed to be critical growth factors in blood: TGF- β, IGF-1, IGF-2, IGFBP-1, IGFBP-2 and IGFBP-3 were measured by a specific enzyme immunoassay with a commercial kit (TGF-β, IGF-1, IGFBP-1, IGFBP-2, IGFBP-3: Duoset®, R&D systems, Minneapolis; IGF-2 : Mediagnost, Reutlingen, Germany; KL-6 level was measured by a specific enzyme immunoassay with a commercial kit (Lumipulse G KL-6 Fujirebio Europe).

### Pulmonary function tests

We performed lung function tests in both routine respiratory laboratory of CHU Liège and Erasme University hospital. All spirometric tests performed for this study were measured using the pneumotachograph JaegerMasterlab system (Erich Jaeger GmbH, Wuzburg, Germany). The forced expiratory volume in one second (FEV1) and forced vital capacity (FVC) were measured in accordance with the recommendations of the European Respiratory Society (ERS) [26]. The results were expressed in millilitre and percent predicted. The Tiffeneau index or FEV1/FVC was expressed in percent. The total lung capacity (TLC) was measured by body plethysmography according to ERS recommendations (Erich Jaeger GmbH, Wuzburg, Germany). The diffusion capacity of CO (DLCO) and the report DLCO/AV were measured by the single-breath carbon monoxide gas transfer method and expressed as percent predicted (Sensor Medics 2400 He/CO Analyzer System, Bilthoven, Netherlands).

### Statistical analysis

Demographic and functional data were expressed as mean ± standard deviation (SD). The biomarkers levels were expressed as median (min-max). When the data showed normal distribution, they were compared with a one-way ANOVA, followed by Tukey-Kramer’s post-hoc testing. When the data did not show a normal distribution, they were compared with the Kruskal-Wallis test followed by Dunn's post-hoc testing. Correlations between variables were performed using Spearman’s rank correlation test. A *p* < 0.05 was considered as significant.

## Results

### Subject demographic functional and blood characteristics

The demographic, functional and treatment characteristics of the subjects are given in Table 1. The average age of IPF patients was slightly higher (74 ± 9 years for untreated patients versus 68 ± 9 years for treated patients) with a male dominance. Spirometric values were moderately lowered and comparable in both the treated and untreated IPF while DLCO was sharply reduced in both IPF groups.

**Table 1:** Patients demographic, functional, treatment and blood characteristics

	Healthy subjects *n* = 55	Untreated IPF *n* = 24	Treated IPF *n* = 26
Age, yrs	60 (8)	74 (9)***	68 (9)***
Gender (M/F)	28/27	19/5	16/3
Height, cm	170 (8)	168 (10)	170 (9)
Weight, Kg	75 (12)	74 (16)	78 (11)
BMI, Kg/m^2^	26 (3)	26 (4)	27 (3)
Smokers (NS/FS/S)	23/22/6	3/8/6	(6/19/0)
Leucocytes x10^3^/μl	7.00 (5.39)	8.35 (2.35)*	8.42 (2.91)**
Neutrophils (Cell/μl)	3450 (1025)	5614 (2113)***	5960 (3053)***
Lymphocytes (Cell/μl)	2085 (620)	1676 (825)*	1786 (643)
Monocytes (Cell/μl)	486 (160)	698 (325)**	725 (218)***
Eosinophils (Cell/μl)	173 (125)	314 (266)**	214 (128)
Basophils (Cell/μl)	33 (17)	55 (67)	94 (187)
Fibrinogen (g/l)	3 (0.80)	^a^4 (1.40)***	nd
CRP (mg/l)	1.9 (2.89)	^a^30 (37.89)***	nd
FEV1 post-BD, %pred.	105 (12)	75 (14)***	67 (13)***
FVC post-BD, %pred.	111 (16)	73 (14)***	66 (16)***
TLC, %pred.	nd	71 (19)	68 (15)
DLCO %pred.	nd	37 (11)	38 (13)
KCO %pred.	nd	63 (16)	70 (20)
Treatment (pirfenidone/nintedanib)	0/0	0/0	17/9
Pirfenidone (duration of treatment -Month)	nd	nd	9.9 (7.4)
Nintedanib (duration of treatment -Month)	nd	nd	14.3 (15.8)

*nd* not determined

Data are expressed as mean (SD)

Non smoker (NS), former smoker (FS), smoker (S)

*p < 0.05 **p < 0.001 ***p < 0.0001 compared to healthy subjects

^a^ *n* = 13

There was a significant increase in leukocyte count of untreated and treated IPF patients (*p* < 0.0001). IPF patients also displayed neutrophil (treated IPF : *p* < 0.0001; untreated IPF : *p* < 0.001), monocyte (treated IPF : *p* < 0.0001; untreated IPF : *p* < 0.0001) and eosinophil counts (treated IPF : *p* > 0.05; untreated IPF : *p* < 0.001) compared to HS (Table 1). By contrast, circulating lymphocyte count was reduced in untreated IPF compared to HS. There was an increase in fibrinogen and CRP levels in untreated IPF compare to HS (Table 1).

### Serum growth factors

**Fig. 2:**
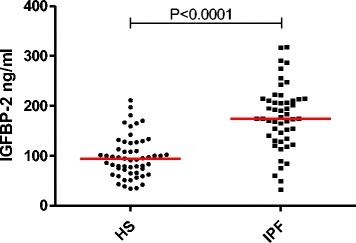
Serum IGFBP-2 concentration: Comparison between IPF and healthy subjects. *HS = healthy subjects (n = 55); IPF = idiopathic pulmonary fibrosis (n = 50: treated and untreated)*

**Fig. 3:**
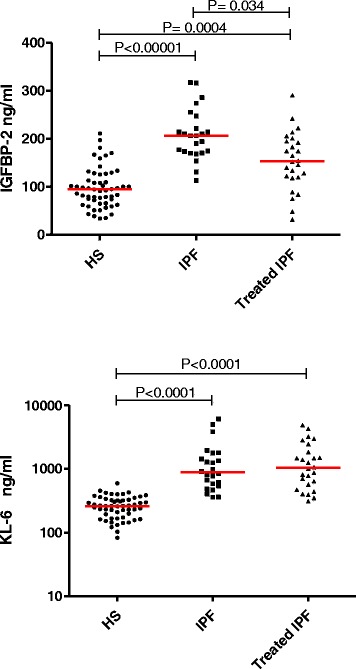
Serum IGFBP-2 and KL-6 concentrations: Comparison between treated IPF, untreated IPF and healthy subjects. *HS = healthy subjects; IPF = idiopathic pulmonary fibrosis*

The results of the serum biochemical markers are listed in the Table 2. There was a striking increase in IGFBP-1 and −2 in the group of untreated IPF patients compared to healthy subjects (respectively *p* < 0.05, *p* < 0.0001) (Figs. 2–3), while untreated IPF showed a significant decrease in serum IGF-1 (*p* < 0.05), IGF-2 (*p* < 0.01) and IGFBP-3 (*p* < 0.01). IGFBP-2 levels were lower in those patients receiving anti-fibrotic therapy but still significantly elevated compared to HS (*p* < 0.001) (Fig. 3). IGFBP-2 seems to be significantly lowered by specific IPF therapy irrespective of the molecule used as anti-fibrotic treatment (median range of 189 (110–231) ng/ml in the group treated by nintedanib (*n* = 9) vs 155 (126–192) ng/ml in those receiving pirfenidone (*n* = 17), *p* > 0.05).

**Table 2:** Serum biomarkers

	Healthy subjects	Untreated IPF	Treated IPF
IGF-1 (ng/ml)	31 (7–67)	20 (9–102)*	25 (11–57)
IGF-2 (ng/ml)	710 (401–2232)	590 (210–1027)*	586 (282–920)**
IGFBP-1 (ng/ml)	11 (0–180)	22 (4–110)*	9 (1–107)
IGFBP-2 (ng/ml)	94 (34–211)	206 (113–317)***	153 (32–291)** °
IGFBP-3 (ng/ml)	2132 (1207–4059)	1536 (534–2556)*	2032 (893–3505)
Molar ratio IGF-1 : IGFBP-1	12 (1–314)	3,5 (0,3–36,3)*	9 (1–152)
Molar ratio IGF-1 : IGFBP-2	1,8 (0,2–6,2)	0,4 (0–4,3)***	0,7 (0,3–5,2)*
Molar ratio IGF-1 : IGFBP-3	0,06 (0,02–0,12)	0,05 (0,03–0,25)	0,05 (0,03–0,1)
Molar ratio IGF-2 : IGFBP-1	247 (20–6735)	110 (11–769)*	259 (20–2429)
Molar ratio IGF-2 : IGFBP-2	40 (10–202)	14 (5–28)***	19 (7–89)**
Molar ratio IGF-2 : IGFBP-3	1,4 (0,8-5,1)	1,6 (0,6-2,2)	1,2 (0,9-1,8)
TGF-β (ng/ml)	11 (3–21)	11 (5–22)	9 (4–17)
KL-6 (ng/ml)	262 (83–596)	1050 (314–4951)***	889 (359–6168)***

Data are expressed as median (min-max)

*p < 0.05 **p < 0.001 ***p < 0.0001 compared to healthy subjects

*°p < 0.05 °°p < 0.001 °°°p < 0.0001 compared to untreated IPF*

Similarly to IGFBP-2, KL-6 was increased in both IPF groups compared to HS (*p* < 0.0001) (Fig. 3). By contrast there was no significant difference between the groups regarding the levels of TGF-β.

We also calculated the serum molar ratio of IGFs:IGFBPs known as reflecting the real IGFs activity (Table 2). Serum ratios of IGF-1:IGFBP-1 (*p* < 0.01), IGF-1:IGFBP-2 (*p* < 0.0001), IGF-2:IGFBP-1 (*p* < 0.05) and IGF-2:IGFBP-2 (*p* < 0.0001) were significantly lower in the not treated IPF group than in healthy subjects (Table 2).

### Relationship between growth factors and lung function

There was an inverse relationship between spirometric values and TGF-β in healthy subjects (FVC % pred *R* = −0.50, *p* < 0.05 and FEV1 % pred *R* = −0.48, *p* < 0.05) and untreated IPF (FEV1 % pred *R* = −0.54, *p* < 0.05) and an inverse relationship between IGFBP-1 and DLCO in treated IPF (*R* = −0.52, *p* < 0.05). We didn’t find any correlation between pulmonary function tests and IGFBP-2 and other biomarkers assessed in our study (Additional file 1: Table S1).

## Discussion

Our study shows for the first time that IPF features a marked increase in serum IGFBP-1 and IGFBP-2. In addition IGFBP-2 levels are attenuated in those patients receiving anti-fibrotic treatment even if the serum levels remained higher in than those measured in healthy subjects.

Previous studies focusing on IGFBP-2 in fibrosis showed an increase of IGFBP-2 in the bronchoalveolar lavage [12] and in the lung tissue of patients with interstitial lung disease (ILD) without focusing on IPF. IGFBP-2 was shown to be overexpressed in case of lung epithelial damage [12–15, 27] and its expression was found to be reduced, together with that of TGF-β, by cyclosporine in vitro [10]. The assay of IGFBP-2 in the serum of patients with idiopathic pulmonary fibrosis had never been done before. In our current study IGFBP-2 was significantly higher in the serum of patients with IPF compared to healthy subjects. Of great interest, serum IGFBP-2 decreases with specific anti-fibrotic treatments without returning to normal values however. It supports the idea that IGFBP-2 may play a role in the fibrotic process. As our patients were not treated with corticoids we can here discard any possible impact of corticosteroids on IGFBP levels [28].

Reinforcing the potential role of IGFBPs in IPF, IGFBP-1 was also specifically increased, but to a lesser extent, in IPF compared to HS. The increase of KL-6 in IPF versus healthy subjects is confirmatory of previous findings [29, 30] and validates our IPF patient cohort. In sharp contrast to what we found with IGFBP, serum IGF-1 and −2 were not found to be decreased in IPF in comparison to HS which results in a decrease ratio IGF:IGFBP in patients with IPF. IGFs are very potent growth factors [20] but, to the best of our knowledge, had not been studied in human lung fibrosis so far. In an animal model IGF-1 was shown to stimulate differentiation of fibroblast into myofibrolast [31], one of the effector of pulmonary fibrosis [32]. Intuitively we might have expected a direct relationship between IGFs and disease severity. However our study didn’t show any correlation between IGF-:IGFBP ratio and functional impairment including forced vital capacity and total lung diffusing capacity. The rise of serum IGFBPs might be seen as a spill over of the lung fibrotic process since previous studies showed raised levels in BAL from patients with ILD [12]. How IGFBP may favour lung fibrosis remains unclear. One potential explanation is IGFBP-2 can bind to the lung extracellular matrix [33] and thus can favour the IGF activity by increasing its local availability, which results in an increase of cellular response to IGF [34]. This trapping mechanism would explain why IGFs themselves were found to be reduced in serum from our patients. Alternatively we cannot rule out the fact that high IGFBP-2 in IPF may actually reflect a protective feed-back mechanism to limit the disease progression by neutralizing IGFs [35]. On the same line there is an example where IGFPB-2 is playing a protective role against anarchic cell proliferation. In non-small cell lung carcinoma, in vitro studies indicate that both soluble and membrane-associated IGFBP-2 competes with IGF receptors for ligand binding and, thus, are likely to be important determinants of IGF responsiveness [36]. Interestingly IGF-I and IGFBP-3 are lowered in untreated IPF patients in comparison to healthy subjects. This decrease is similar to what it has been shown in ARDS, cystic fibrosis and COPD [37]. Moreover these authors suggest that there may be an inverse association between circulating levels of IGF-1 and IGFBP-3 and lung compartments levels where they are upregulated suggesting a different lung and blood regulation under these circumstances.

In our study serum levels of TGF-β were similar between the groups confirming the results described by a previous study [38]. Of course it does not mean that these molecules did not play a major role within the lung. Indeed TGF-β is known to take part to the lung fibrosing process and was shown to be overexpressed in lung tissue of patients with IPF [39].

## Conclusion

We conclude that serum IGFBP-1 and IGFBP-2 are increased in patients with idiopathic pulmonary fibrosis in comparison to healthy subjects. Moreover, the raised IGFBP-2 level is attenuated by anti-fibrotic treatment. The prognostic value of these new biomarkers warrants further longitudinal studies.

## Additional file

Additional file 1: Table S1. Correlations between blood biomarkers and pulmonary function test. (DOCX 173 kb)

## Abbreviations

ATS: American thoracic society
AV: Alveolar ventilation
CCL-18: chemokine (C-C motif) ligand 18
COPD: chronic obstructive pulmonary disease
DLCO: diffusion capacity of CO in the lung
FEV1: forced expiratory volume in one second
FVC: forced vital capacity
HRCT: high resolution computed tomography
IGF: insulin-like growth factor
IGFBP: insulin-like growth factor binding protein
IGFBP-rp: insulin-like growth factor binding protein related protein
ILD: interstitial lung disease
IPF: idiopathic pulmonary fibrosis
KL-6: Krebs von den Lungen-6
MMP: matrix metalloproteinase
NSIP: non specific idiopathic pulmonary fibrosis
SD: standard deviation
SP-A: surfactant protein-A
SP-D: surfactant protein-D
TGF-β: transforming growth factor beta
TLC: total lung capacity
YKL-40: Chitinase-3-like-1, human cartilage glycoprotein-39
