# Supplementary material for: Raised serum levels of IGFBP-1 and IGFBP-2 in idiopathic pulmonary fibrosis
Source: BMC Pulm Med. 2016 May 23;16:86. doi: 10.1186/s12890-016-0249-6 (PMC4877734; doi:10.1186/s12890-016-0249-6)
Supplement: Additional file 1: Table S1. — Correlations between blood biomarkers and pulmonary function test. (DOCX 173 kb) [file 12890_2016_249_MOESM1_ESM.docx]

**Additional file 1: Table S1**

**Correlations between blood biomarkers and pulmonary function test**

|  |  | **FEV-1 (%)** | | | **FVC (%)** | | | **DLCO (%)** | | |
| --- | --- | --- | --- | --- | --- | --- | --- | --- | --- | --- |
|  |  | **HS** | **IPF** | **Treated IPF** | **HS** | **IPF** | **Treated IPF** | **HS** | **IPF** | **Treated IPF** |
| **IGF-1** | p value | **0,0073** | 0,7101 | 0,5217 | 0,0322 | 0,8931 | 0,7938 | nd | 0,3487 | 0,3035 |
|  | R= | **0,46** | 0,09 | -0,14 | 0,38 | 0,03 | -0,06 | nd | -0,23 | -0,22 |
| **IGF-2** | p value | 0,223 | 0,5018 | 0,5364 | 0,3139 | 0,8963 | 0,4935 | nd | 0,6349 | 0,5775 |
|  | R= | 0,25 | 0,16 | -0,13 | 0,21 | -0,03 | -0,14 | nd | -0,12 | -0,12 |
| **IGFBP-1** | p value | 0,2199 | 0,3906 | 0,2801 | 0,0324 | 0,0386 | 0,6578 | nd | 0,3061 | **0,0116** |
|  | R= | 0,22 | 0,21 | -0,23 | 0,38 | -0,49 | -0,09 | nd | -0,25 | **-0,52** |
| **IGFBP-2** | p value | 0,2081 | 0,1711 | 0,6874 | 0,1801 | 0,2354 | 0,8493 | nd | 0,8464 | 0,6965 |
|  | R= | 0,23 | -0,33 | -0,09 | 0,24 | -0,29 | -0,04 | nd | 0,05 | -0,09 |
| **IGFBP-3** | p value | 0,199 | 0,0983 | 0,6688 | 0,0282 | 0,2518 | 0,9214 | nd | 0,7634 | 0,3178 |
|  | R= | 0,23 | -0,36 | 0,09 | 0,39 | -0,26 | -0,02 | nd | 0,07 | -0,22 |
| **Ratio  IGF-1:IGFBP-1** | p value | 0,4242 | 0,6059 | 0,4511 | 0,1186 | 0,5013 | 0,5219 | nd | 0,0879 | 0,2979 |
|  | R= | -0,14 | -0,13 | 0,16 | -0,28 | 0,17 | 0,13 | nd | 0,40 | 0,23 |
| **Ratio  IGF-1:IGFBP-2** | p value | 0,9996 | 0,1562 | 0,6889 | 0,769 | 0,3419 | 0,8179 | nd | 0,7087 | 0,8614 |
|  | R= | 0,00 | 0,34 | 0,09 | -0,05 | 0,24 | 0,05 | nd | -0,09 | 0,04 |
| **Ratio  IGF-1:IGFBP-3** | p value | 0,0608 | 0,0385 | 0,6357 | 0,2943 | 0,0891 | 0,917 | nd | 0,4477 | 0,9126 |
|  | R= | 0,33 | 0,44 | -0,10 | 0,19 | 0,38 | -0,02 | nd | 0,17 | 0,02 |
| **Ratio  IGF-2 : IGFBP-1** | p value | 0,5747 | 0,4119 | 0,8511 | 0,105 | 0,6091 | 0,9243 | nd | 0,5977 | 0,7099 |
|  | R= | -0,12 | -0,18 | -0,04 | -0,34 | -0,12 | 0,02 | nd | 0,12 | -0,08 |
| **Ratio  IGF-2 : IGFBP-2** | p value | 0,3184 | 0,3015 | 0,519 | 0,4169 | 0,2371 | 0,5787 | nd | 0,7493 | 0,802 |
|  | R= | -0,21 | 0,25 | 0,14 | -0,17 | 0,29 | 0,12 | nd | 0,08 | 0,06 |
| **Ratio  IGF-2 : IGFBP-3** | p value | 0,6823 | 0,8633 | 0,6637 | 0,8852 | 0,7238 | 0,9185 | nd | 0,7905 | 0,55 |
|  | R= | 0,09 | 0,04 | -0,09 | 0,03 | 0,08 | 0,02 | nd | 0,06 | 0,13 |
| **KL-6** | p value | 0,2257 | 0,4189 | 0,5423 | 0,1978 | 0,6074 | 0,486 | nd | 0,6808 | 0,8886 |
|  | R= | 0,22 | 0,20 | -0,13 | 0,24 | -0,13 | -0,15 | nd | -0,10 | 0,03 |
| **TGF-β** | p value | **0,0219** | **0,011** | 0,9026 | **0,0143** | 0,1653 | 0,6023 | nd | 0,1986 | 0,2294 |
|  | R= | **-0,48** | **-0,54** | -0,03 | **-0,50** | -0,32 | 0,11 | nd | -0,29 | 0,27 |
